# Supplementary material for: Computing nasalance with MFCCs and Convolutional Neural Networks
Source: PLoS One. 2024 Dec 31;19(12):e0315452. doi: 10.1371/journal.pone.0315452 (PMC11687758; doi:10.1371/journal.pone.0315452)
Supplement: Attachment — Submitted filename: Response_to_reviewers.pdf [file pone.0315452.s002.pdf]

Response to Reviewer 3 Comments

Comments made by the reviewer have been applied to correct erroneous terminology used, complement the referenced bibliography, and clarify parts of the paper for better understanding.

Questions

Question 1: Abstract, Table 1, elsewhere: "The test data were recorded in Spain and included short words (-dynamic), sentences (+dynamic), and diakinetic syllables (+dynamic)"

-What do these +/- labels mean?

- Why is the /ka/ in "ka" considered "dynamic" but the /ka/ in "boka" isn't? These terms are never defined or justified.

- I am not familiar with the term "diakinetic". Do you mean "diadochokinetic"?

Response 1: Label means: more (+) and less (-). Abstract is modified to include clarification.

Utterance in Table 1, Diakinetic syllable repetition is modified. Instead of /ka/ is marked as /kakaka…/, and a paragraph is added to clarify that it represents many repetitions of syllable /ka.

The term diakinetic is replaced by diadochokinetic.

Question 2: p.3: "To our knowledge, only one ML model has the flexibility of Nasometry [18]"

-The authors may want to be aware of similar works that have previous been carried out, for example Siriwardena et al. (2024) and Carignan (2021), the latter of which has been extended to low-resource languages (Carignan et al., 2023) and hyper-nasality in children (Fagniart et al.,2024), two goals which the current study also aims to meet.

Carignan, C. (2021). "A practical method of estimating the time-varying degree of vowel nasalization from acoustic features", J. Acoust. Soc. Am. 149 (2), 911–922. doi: https://doi.org/10.1121/10.0002925

Carignan, C., Chen, J., Harvey, M., Stockigt, C., Simpson, J. & Strangways, S. (2023). "An investigation of the dynamics of vowel nasalization in Arabana using machine learning of acoustic features", Laboratory Phonology 14(1). doi: https://doi.org/10.16995/labphon.9152

Fagniart, S., Charlier, B., Delvaux, V., Huberlant, A., Harmegnies, B.G., Piccaluga, M., & Huet,K. (2024). "Consonant and vowel production in children with cochlear implants: acoustic measures and multiple factor analysis", Front. Audiol. Otol. 2:1425959. doi:10.3389/fauot.2024.1425959

Siriwardena, Y.M., Boyce, S.E., Tiede, M.K., Oren, L., Fletcher, B., Stern, M., & Espy-Wilson,C.Y. (2024). "Speaker-independent speech inversion for recovery of velopharyngeal port constriction degree", J. Acoust. Soc. Am. 156 (2), 1380–1390. doi: https://doi.org/10.1121/10.0028124

Response 2: These related works have been added in the Introduction section.

Question 3: You say that European Spanish is "a non-nasal dialect" (p.3) and that "Costa Rican Spanish is known to be highly nasal, while European and Chilean Spanish are comparatively similarly oral"(p.15), but how do these statements align with the statement that "vowels in contact with nasal consonants tend to be nasalized (universally)" (p.2)?

Response 3: The fact that vowels in contact with a nasal tend to nasalise is universal, although dialects and languages vary in the degree to which this occurs, as is in the case of European and Chilean Spanish.

Changes in text are added: "However, because vowels in contact with nasal consonants tend to be nasalized (universally, although the degree varies according to language or dialect), they used these vowel fragments to create a large set of nasal vowels".

Question 4: p.4: "These signals are divided into 250 ms windows with 100 ms overlap (i.e., every window starts 100 ms after the previous one)"-Based on this description, it would seem that every window starts 150 ms (not 100 ms) after the previous one.

Response 4: Every window starts 100 ms after the previous one. Text modified to be accurate to 150 ms overlap.

Question 5: p.6: "(/m/, /n/ and /ñ/)"

-"/ñ/" is not a symbol used in the IPA to denote any known consonant.

Response 5: /ñ/ is replaced by the IPA symbol /ɲ/ .

Question 6: p.6: "In this study, we assumed that the entire vowel was nasalized if it was shorter than 60 ms, 50% if it was between 60 and 90 ms, and 30% if it was longer than 90 ms."

-Based on what evidence?

Response 6: This distribution of vowel nasalisation length is based on experiments conducted in the early design phases of the present study. A subset of the training group is selected and trained with an automatic classification system, the aim being to select different nasalised vowel lengths so that the output corresponds to the annotation produced by the speech specialist. The results shown in the paper are the ones that obtain an optimal result.

Text is modified to add clarification.

Question 7: p.7: "The transformation from a percentage to a 4-level scale required deciding the limits among classes. As we knew in advance how many children were classified perceptually as oral or nasal, or as Oral, Mild, Moderate or Severely nasal, we assumed that the same numbers would apply in the case of nasalance measures. Thus, after every simulation, the results were sorted per mfccNasalance, with the first N cases being categorized as Healthy, the next M cases as Mild, etc."

-This should be explained much more clearly, I don't understand this at all. Does this just meant that you assigned identical labels as the perceptual labels?

Response 7: The CNN output produces values between 0 and 1, which have no clinical value. Therefore, the output is transformed into a 4-value scale. This transformation is done keeping the original training case distribution, from which we know the classification as oral, mild, moderate or severely nasal.

Text has been modified to clarify it.

Question 8: p.9: "Finally, the percentage ratings were recoded using a 4-level scale. Oral: nasality ≤ 0.05;Mild: 0.05 < nasality ≤ 0.25; Moderate: 0.25 < nasality ≤ 0.50; Severe: nasality > 0.50."

-Based on what evidence?

Response 8: This measure is supported by clinical practice experience in our lab. Experience shows that:

- Any healthy speaker can nasalize occasionally (hence 5% seems a reasonable limit).

- Nasalizing over 50% of the speech utterances is evidence of severe hypernasality.

- Intervals between 5% and 50% are divided equally.

A clarification is added in this section.

Question 9: p.9: "Nasalance was computed as the ratio of nasal acoustic energy to the sum of oral and nasal energy".

-Please specify that you used RMS energy, as shown in the Praat script.

Response 9: RMS energy use is specified.

Question 10: Fig.6, etc.: Why not two sets of box plots, or two sets of violin plots, etc.? Why one box plot and one line?

Response 10: The line represents the eNasalance correlation with the perceptual nasality scores of human experts, it only has one measure, that's why is represented as a line.

The boxplots represent the mfccNasalance correlation with human experts. They have 126 results per utterance type, as we made a k-fold = 5 cross-correlation with 126 kernel combinations and compute the mean value for kernel.

The main purpose of Figure 6 was to illustrate the correlation between eNasalance and human experts, and mfccNasalance with human experts. Separate images are created to illustrate the cases with all available data (left), while the figure on the right shows the cases with the optimal configuration: Spain: Syllable k11 = True, Words Kernels = Temporal, Sentences k11 = True.

Question 11: Fig.6, etc.: The caption says, "Correlation between e-Nasalance [...] and mfccNasalance", but this is not accurate. They are separate correlations between e-Nasalance and perceptual scores, and mfccNasalance and perceptual scores.

Response 11: Fig. 6 and 7 captions are modified to include: "Correlation between e-Nasalance and perceptual scores (orange rectangle), and mfccNasalance and perceptual scores (blue)"

Question 12: p.10: "the scores are higher than eNasalance in most cases (i.e. all except the lower 25thpercentile)."

-Are you referring to the lower whisker? The whisker is not the 25th percentile.

Response 12: Sentence is modified to indicate: "i.e. all except the lower whisker".

Question 13: Table 2: Do you mean "p" instead of "s"? Also, what is "k11"?

Response 13: "s" is changed to "p".

k11 denoted the presence of a 1 × 1 kernel in the first layer of the CNN model. Clarification added in Table 2 and Table 3.

Question 14: p.13: "(Fig. 7 down right)" -> "bottom right"

Response 14: Text is corrected.

Question 15: p.14: "These results are compatible with the fact that nasalance is a poor approximation to perceptual nasality"

-Citation needed. Nasalance arose as a methodology precisely because it approximates perceptual nasality.

Response 15: Citation is added. Studies comparing the nasalance scores and perceptual scores have obtained correlations ranging from non-significant to strong.

Liu, Y.; Lee, S.A.S.; Chen,W. The correlation between perceptual ratings and nasalance scores in resonance disorders: A systematic review. J. Speech Lang. Hear. Res. 2022, 65, 2215–2234. [CrossRef] [PubMed]

Question 16: p.14: "in contrast, as we only need to know that a nasal sound was present (and not the exact location)"

-Maybe I've misunderstood, but this doesn't seem correct, because you based your labels off of phone-wise segmentation. In order to have phone-wise segmentation, you need to know the exact location of that phone in the speech stream.

Response 16: In contrast to Mathad et al., who use a 25 ms window, the present work uses a CNN with a larger window, 250 ms, which allows for less accurate annotation.

In the present work the classification of a sound as oral/nasal is based on the percentage of nasality present, so an error in the precise location of the nasal sound does not prevent the 250ms fragment from being classified correctly.

A clarification is added in this section.

Question 17: Punctuation errors throughout.

Response 17: Punctuation errors are corrected.
