# Supplementary material for: Computing nasalance with MFCCs and Convolutional Neural Networks
Source: PLoS One. 2024 Dec 31;19(12):e0315452. doi: 10.1371/journal.pone.0315452 (PMC11687758; doi:10.1371/journal.pone.0315452)
Supplement: Attachment — Submitted filename: Response_to_reviewers.docx [file pone.0315452.s001.docx]

**Journal requirements:**

When submitting your revision, we need you to address these additional requirements.

1. Please ensure that your manuscript meets PLOS ONE's style requirements, including those for file naming. The PLOS ONE style templates can be found at

<https://journals.plos.org/plosone/s/file?id=wjVg/PLOSOne_formatting_sample_main_body.pdf>

<https://journals.plos.org/plosone/s/file?id=ba62/PLOSOne_formatting_sample_title_authors_affiliations.pdf>.

**1:** Manuscript have been modified to meet PLOS ONE’s style requirements..

2. Please note that PLOS ONE has specific guidelines on code sharing for submissions in which author-generated code underpins the findings in the manuscript. In these cases, we expect all author-generated code to be made available without restrictions upon publication of the work. Please review our guidelines at https://journals.plos.org/plosone/s/materials-and-software-sharing#loc-sharing-code and ensure that your code is shared in a way that follows best practice and facilitates reproducibility and reuse."

**2:** All autor-generated code script files can be found in public repository:

https://doi.org/10.6084/m9.figshare.26762818.v1

https://doi.org/10.6084/m9.figshare.26762842.v1

https://doi.org/10.6084/m9.figshare.26762833.v1

3. Thank you for stating the following financial disclosure: [This research was funded by the Spanish MINISTERIO DE CIENCIA, INNOVACIÓN Y UNIVERSIDADES, grant number PID2021-126366OB-I00.]. Please state what role the funders took in the study. If the funders had no role, please state: ""The funders had no role in study design, data collection and analysis, decision to publish, or preparation of the manuscript."" If this statement is not correct you must amend it as needed. Please include this amended Role of Funder statement in your cover letter; we will change the online submission form on your behalf.

**3:** Added “The funders had no role in study design, data collection and analysis, decision to publish, or preparation of the manuscript.” in the financial disclosure.

4. We note that you have indicated that there are restrictions to data sharing for this study. PLOS only allows data to be available upon request if there are legal or ethical restrictions on sharing data publicly. For more information on unacceptable data access restrictions, please see http://journals.plos.org/plosone/s/data-availability#loc-unacceptable-data-access-restrictions.

Before we proceed with your manuscript, please address the following prompts:

a) If there are ethical or legal restrictions on sharing a de-identified data set, please explain them in detail (e.g., data contain potentially identifying or sensitive patient information, data are owned by a third-party organization, etc.) and who has imposed them (e.g., a Research Ethics Committee or Institutional Review Board, etc.). Please also provide contact information for a data access committee, ethics committee, or other institutional body to which data requests may be sent.

b) If there are no restrictions, please upload the minimal anonymized data set necessary to replicate your study findings to a stable, public repository and provide us with the relevant URLs, DOIs, or accession numbers. For a list of recommended repositories, please see https://journals.plos.org/plosone/s/recommended-repositories. You also have the option of uploading the data as Supporting Information files, but we would recommend depositing data directly to a data repository if possible.

We will update your Data Availability statement on your behalf to reflect the information you provide.

**4:** Minimal anonymized data set can be found in public repository:

https://doi.org/10.6084/m9.figshare.26762818.v1

https://doi.org/10.6084/m9.figshare.26762842.v1

https://doi.org/10.6084/m9.figshare.26762833.v1

5. Your ethics statement should only appear in the Methods section of your manuscript. If your ethics statement is written in any section besides the Methods, please delete it from any other section.

**5:** Ethics statement only appear in Method section.

6. Please upload a copy of Supplementary files to which you refer in your text on page 12. Please amend the file type to 'Supporting Information'. If the Supplementary file is no longer to be included as part of the submission, please remove all reference to it within the text.

**6:** Supplementary files reference has been removed within the text.

Response to Reviewer 1 Comments

Comments made by the reviewer have been applied to improve the reliability of the results and the robustness of the methods applied. In particular, the use of 39MFCC, the cross-validation of the data, and an improvement in the depth of the statistical analysis have led to a substantial change in the results obtained.

Therefore, in this revised version of the article, extensive changes have been made in the Results and Discussion sections, modifying the images generated and the way of showing and justifying the results obtained.

**Questions**

**Question 1:** Why the center frequency reduced to 500Hz (in method) instead of 600Hz according to the reference [5, 7] as mentioned in the Introduction?

**Response 1:** Changed to: “600 Hz” in Methods.

**Question 2:** According to the method described by Mathad et al. (2021), MFCCs and their first and second deltas were utilized to train the DNN. In contrast, your method includes only MFCCs without the deltas, which requires further explanation. [if there is no justification provided, please implement R2]

**Response 2:** In the present version, training is run using 39 MFCCs coefficient with delta and second-delta in the method section, as suggested by both reviewers.

**Revisions**

**Revision 1:** In comparison to the reference study by Mathad et al. 2021, which utilized a substantial 100-hour database, the training model in that study is robust enough to mitigate any subset effects. However, the current paper relies on only 2 hours of data without employing cross-validation. To enhance the reliability of the results, I recommend implementing cross-validation and conducting a straightforward comparison of accuracy (%) means to strengthen the statistical validity of the findings.

**Response 1:** Cross-validation process has been implemented for train and validation speakers.

**Revision 2:** The deltas of MFCCs are crucial, as demonstrated in numerous studies, for capturing the temporal dynamics that are often lost during time windowing. By including the deltas, the current spectral dimension would be expanded to a three-dimensional matrix: 2 (channels) × 39 MFCC features (13 MFCCs + 13 first deltas + 13 second deltas) × 26 timeframes. This addition would enhance the model's ability to recover temporal information.

**Response 2:** Thanks for your suggestion. 39 MFCCs coefficient with delta and second-delta are computed to generate a 2-channel image 2 (channels) × 39 MFCC × 26 timeframes as input data of CNN.

**Typos**

**Typos 1:** Ensure that all graphs include labels on the y-axis. Currently, they are missing.

**Response 1:** Graphs has been modified to include y-axis labels.

**Typos 2:** Address the numerous typos throughout the paper, with particular attention to consistent capitalization and formatting of "MFCCs."

**Response 1:** Typos corrected to use consistent “MFCCs”

Response to Reviewer 2 Comments

**Questions**

**Question 1:** The utilization of CNN on MFCC for classification and nasal and oral radiated energy-based classification are well-established. So, the article has lack of novelty. The description of the optimized kernel is confusing. The author should represent the kernel with respect to individual MFCC and their derivatives. The word and phrase selection should be unified. For an example, the spectrogram and MFCC are used same purpose, which made the reader confused.

**Response 1:**  The description of the optimized kernel has been modified in the Results section.

MFCCs features with delta and second delta has been computed and added as input of CNN.

Word and phrase selection remains unchanged because the results show different behaviour in terms of the optimal kernel configuration for each type of utterance, which we consider worthy of distinction and discussion.

Spectrogram and MFCC are no longer used for the same purpose. Input data generated with MFCCs coefficients are defined as ‘MFCCs input images’ in the text.
