# Supplementary material for: Computing nasalance with MFCCs and Convolutional Neural Networks
Source: PLoS One. 2024 Dec 31;19(12):e0315452. doi: 10.1371/journal.pone.0315452 (PMC11687758; doi:10.1371/journal.pone.0315452)
Supplement: Attachment — Submitted filename: Response_to_reviewers.docx [file pone.0315452.s003.docx]

Response to Reviewer 3 Comments

New comments made by the reviewer have been applied to correct issues that remain from previous review. Minor typos are corrected, and additional text has been added.

**Questions**

**Question 1:**  p.2: "Furthermore, there are currently multiple machine learning (ML) algorithms that may serve to classify complex feature sets, such as MFCCs (see [12-14]), or high-speed nasopharyngoscopy [15]."

-The authors seem to have missed the point in providing these additional references, which are not simply for "classify[ing] complex feature sets".

Carignan (2021) uses MFCCs in combination with other acoustic features to train an ML algorithm to create nasalance-like signals and compares the results with nasometry measures.

Siriwardena et al. (2024) uses the full acoustic waveform to train an ML algorithm to create nasalance-like signals and compare the results with nasopharyngoscopy.

Therefore, both of these works have previously used speech acoustics to train ML algorithms to create nasalance-like signals, in a similar manner to Mathad et al. (2021) and to the current study, which is somewhat at odds with the claim that "only one ML model has the flexibility of Nasometry" (p. 2); both Carignan (2021) and Siriwardena et al.(2024) were carried out very specifically to generate nasalance-like signals from speech acoustics using ML algorithms.

**Response 1:** Thank you for the clarification, text has been adapted to the suggestions mentioned above.

Text added in section “Previous ML proposals”:

Other studies have adopted flexible approaches. Carignan et al. [14] uses MFCCs in combination with other acoustic features to train an ML algorithm to create nasalance-like signals and compares the results with Nasometry measures. However, they do not analyse the interest of this approach for speech assessment, which is the main aim of this study. Siriwardena et al. [15] uses the full acoustic waveform to train an ML algorithm to model nasalance and compare the results with nasopharyngoscopy. However, in speech therapy, both Nasometry and nasopharyngoscopy are instrumental methods, whose validity needs to be compared with the ground truth (i.e. perceptual evaluation; [1, 23]). In contrast to all other models, Mathad et al. [22] trained their model, a Deep Neural Network (DNN), exclusively with speech samples from healthy speakers, without any clinical data, and aim to measure hypernasality in children with HN pathology.

**Question 2:** Response 13: “s” is changed to “p”

-"s" remains in the text at many different points

**Response 2:** The remaining instances of “s” have been changed to “p”.
